# Expression of HE4 in Endometrial Cancer and Its Clinical Significance

**DOI:** 10.1155/2015/437468

**Published:** 2015-10-11

**Authors:** Xiao Li, Yiping Gao, Mingzi Tan, Huiyu Zhuang, Jian Gao, Zhenhua Hu, Huimin Wang, Liancheng Zhu, Juanjuan Liu, Bei Lin

**Affiliations:** Department of Obstetrics and Gynecology, Shengjing Hospital of China Medical University, Shenyang, Liaoning 110004, China

## Abstract

The main aims of this study were to determine the expression of human epididymis protein 4 (HE4) in endometrial cancer and to explore the relationships between HE4 expression, clinicopathological parameters, and prognosis. Immunohistochemistry was used to detect HE4 expression in 102 cases of endometrial cancer, 30 cases of endometrial atypical hyperplasia, and 20 cases of normal endometrium. The positive expression rate of HE4 in endometrial carcinoma was 84.62%, significantly higher than 66.67% in atypical hyperplasia (*P* < 0.05) and 15.00% in normal endometrium (*P* < 0.0.01). With the exception of stage II, HE4 expression in endometrial cancer showed an increasing tendency with increased clinical stage (*P* < 0.05). The positive expression rate of HE4 increased with a decrease in the degree of differentiation. A statistically significant difference was observed between the highly differentiated group and the poorly differentiated group (*P* < 0.05). Mortality in endometrial cancer patients with high HE4 expression was significantly higher than that in patients with low HE4 expression (*P* < 0.05). Endometrial cancer patients with high HE4 expression have a poor prognosis.

## 1. Introduction

Endometrial cancer is a malignant cancer with endometrial epithelial origin, accounting for 20% to 30% of malignant tumors in the female reproductive system. In recent years, due to increased obesity, hypertension, diabetes, and prolonged life expectancy, the incidence and mortality of endometrial cancer have risen, with a tendency for onset at a younger age [1]. The development of endometrial cancer is a multifactorial and multistep process. With an early manifestation of vaginal bleeding after menopause, approximately 70% of patients are diagnosed by fractional curettage at an early stage. However, the remaining 30% of patients with high risk factors are diagnosed with endometrial cancer at an advanced stage [2]. Thus, increasing the rate of early diagnosis is not only an important way of improving prognosis but also the key to increasing overall survival in patients with endometrial cancer. Up to now, no serum tumor markers with high sensitivity and specificity have been identified. Human epididymis protein 4 (HE4) is also known as whey acidic protein (WFDC2). In 1999, Schummer et al. [3] first observed HE4 overexpression in ovarian cancer tissue. In 2003, HE4 was approved by the FDA as a serum tumor marker for ovarian cancer and attracted great attention [4]. The detection of HE4 alone or combined with CA125 can improve the diagnostic sensitivity and specificity of epithelial ovarian cancer [5, 6]. Recent studies have shown that HE4 is highly expressed in ovarian cancer tissue, as well as in other malignant tumors including lung adenocarcinoma, stomach cancer, and pancreatic cancer [7, 8]. In 2011, Yang et al. [9] performed an immunohistochemical assay and enzyme-linked immunosorbent assay to determine HE4 expression in tissue and peripheral blood from 31 cases of endometrial cancer for the first time, and the results indicated that the positive expression rate of HE4 and serum level of HE4 in the malignant group were significantly higher than those in the normal endometrium group (20 cases) and the endometrial hyperplasia group (19 cases), and the difference was statistically significant.

Our previous studies showed that high expression of HE4 was observed in ovarian cancer [10] and the positive expression rate in fallopian tube cancer was significantly higher than that in normal fallopian tube tissue [to be published]. The uterus, fallopian tubes, and ovaries originate from the urogenital ridge, and the former two originate from the paramesonephric duct; thus they possess similar embryogenic properties. Therefore, based on the above-mentioned theory, this study detected the expression of HE4 in endometrial cancer, endometrial atypical hyperplasia, and normal endometrium tissue samples with an adequate sample size and explored the relationship between HE4 expression and histological type, stage, differentiation, and prognosis of endometrial cancer, in order to provide a theoretical basis for an in-depth mechanism study of endometrial cancer development.

## 2. Materials and Methods

### 2.1. Patients

The paraffin-embedded samples examined in this study were collected during surgery from 173 cases treated in the Department of Obstetrics and Gynecology, Shengjing Hospital of China Medical University from 2004 to 2013. The pathological diagnosis of all tissue sections was determined by experts from the Department of Pathology, Shengjing Hospital of China Medical University. Of these 173 cases, there were 102 cases of endometrial cancer, 30 patients with atypical endometrial hyperplasia (10 cases each in the severe, moderate, and mild subgroups), and 20 patients with normal endometrium (10 cases each in the secretory and proliferative phase, resp.). Normal endometrium was donated by females with no fertility requirements, who underwent hysterectomy or removal of the uterus plus double annex due to cervical lesions. Enrollment criteria specifically excluded patients with uterine fibroids, ovarian cysts, and other uterine or ovarian diseases. Endometrial cancer patients were aged between 31 and 79 years old, mean 58.09 years; the patients with atypical endometrial hyperplasia were aged between 30 and 66 years old, mean 44.67 years; the patients with normal endometrium were aged between 34 and 53 years old, mean 44.50 years. The difference in age between the groups was not statistically significant (*P* > 0.05). In the endometrial cancer group, the pathological types consisted of 49 cases of endometrial adenocarcinoma, 22 cases of papillary serous adenocarcinoma, 21 cases of clear cell carcinoma, and 10 cases with other pathological types (including mucinous carcinoma, squamous cell carcinoma, undifferentiated cancer, and small cell carcinoma). With regard to the histological grade, there were 23 cases of highly differentiated cancer, 21 cases of moderately differentiated cancer, and 51 cases of poorly differentiated cancer. According to the staging of the International Federation of Gynecology and Obstetrics (FIGO) in 2009, there were 61 cases at stage I (38 cases at stage Ia and 23 cases at stage Ib), 7 at stage II, 28 at stage III, and 6 at stage IV. There were 27 patients with lymph node metastasis and 59 without lymph node metastasis. All patients had primary endometrial cancer, with complete clinical and pathological data and received no preoperative chemotherapy or hormone therapy.

### 2.2. Immunohistochemistry

Histologic sections of each group of fallopian tube tissues were 5 *μ*m. The pattern of expression of HE4 in endometrial carcinoma tissues was analyzed via immunohistochemical streptavidin-peroxidase staining. Positive and negative immunohistochemistry controls were used. Ovarian carcinoma tissue served as a positive control. The negative control was incubated with phosphate-buffered saline instead of primary antibody. The working concentrations of primary antibodies against HE4 were 1 : 400 (rabbit polyclonal anti-HE4 antibody; Abcam, Cambridge, UK). The empirical procedure was performed based on the manufacturer's instructions.

### 2.3. Assessment Criteria

Immunohistochemical staining results, brown-stained granules on the cell membrane and cytoplasm, were regarded as positive. Based on the strength of color, uncolored, light yellow, yellowish brown, and brown were scored as 0, 1, 2, and 3, respectively. The percentage of stained cells in the field of view was calculated as follows: five consecutive high-powered fields in each section were observed under a 400x optical microscope, and then the scores were averaged. The proportion of positive cells <5% was recorded as 0, 5%–25% as 1, 21%–50% as 2, 51%–75% as 3, and >75% as 4. The final score was equal to the multiplication of the two scores: 0–2 as negative (−), 3-4 as weakly positive (+), and 5–12 as strongly positive (2+/3+) expression. For error control, the pathological section was evaluated by two observers separately; if any disagreement occurred, the result was judged by the third pathologist.

### 2.4. Statistical Analysis

Using the SPSS17.0 software system, *χ* ^2^ test and Fisher exact test were conducted. The* t*-test was used for comparisons between two groups and analysis of variance for multiple group comparisons. Kaplan-Meier analysis and the log-rank test were applied for the survival curve. *P* < 0.05 was considered statistically significant.

## 3. Results

### 3.1. HE4 Expression in Endometrial Tissue

HE4 was mainly expressed in the cell membrane, and the cytoplasm also showed slight expression. The positive expression rate of HE4 was 84.62% in the endometrial cancer group, significantly higher than 66.67% in the endometrial atypical hyperplasia group, and 15.00% in the normal endometrium group (*P* = 0.014, 0.001). The positive expression rate of HE4 was 40.00% in the mild atypical hyperplasia group, 80.00% in the moderate atypical hyperplasia group, and 80.00% in the severe atypical hyperplasia group, respectively. The positive rate of HE4 expression in the moderate and severe atypical hyperplasia groups was significantly higher than that in the mild atypical hyperplasia group (*P* = 0.045) and normal endometrium group (*P* < 0.001). The positive expression rate of HE4 in the proliferative phase was higher than that in the secretory phase (20% versus 10%), but there was no significant difference between them (*P* > 0.05).

**Figure 1:**
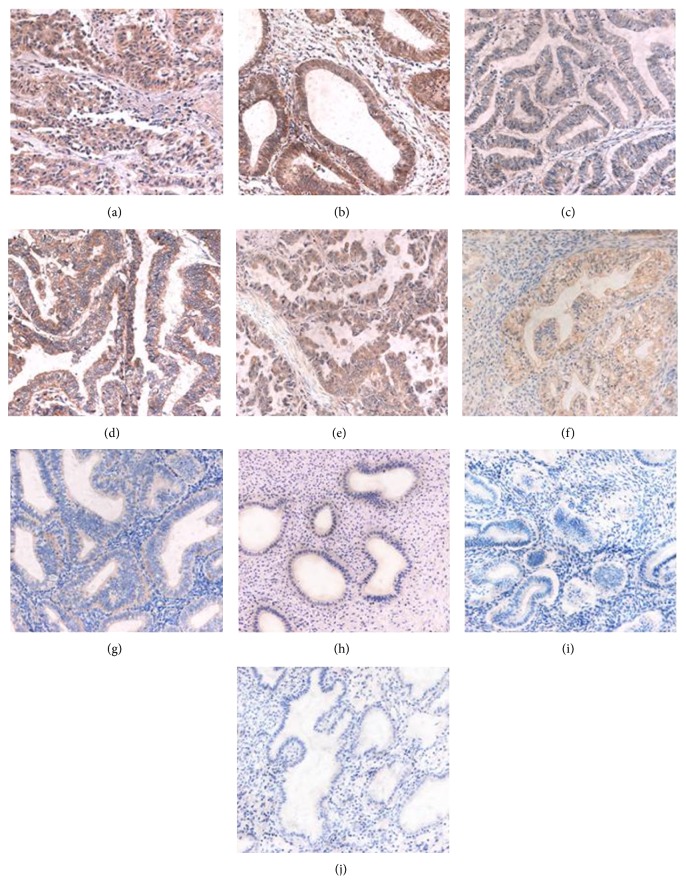
Immunohistochemical micrographs of HE4 in different endometrial tissues (200x). The expression level of HE4 was higher in endometrial cancer than in endometrial atypical hyperplasia and normal endometrium. (a) Poorly; (b) moderately; and (c) highly differentiated adenocarcinoma. (d) Clear cell carcinoma and (E) uterine papillary serous carcinoma. (f) Severe; (g) moderate; and (h) mild atypical hyperplasia. (i) Secretory phase and (j) proliferative phase normal endometrium.

HE4 expression intensity increased with increased degree of malignancy. The strongly positive (2+/3+) expression rate in endometrial cancer was 55.98%, significantly higher than that in the atypical hyperplasia group (20.00%) (*P* = 0.003), and in the normal endometrium group (0.00%) (*P* = 0.033). The strongly positive expression rate of HE4 was 0.00% in the mild atypical hyperplasia group, 20.00% in the moderate atypical hyperplasia group, and 40.00% in the severe atypical hyperplasia group, respectively, which showed an increasing tendency with aggregation of the disease. The strongly positive expression rate of HE4 in the severe hyperplasia group was significantly higher than that in the mild hyperplasia group (*P* = 0.025, <0.05). The strongly positive expression rate of HE4 in the moderate and severe atypical hyperplasia groups was significantly higher than that in the normal endometrium group (*P* = 0.031, 0.002, all *P* < 0.05) (Figure 1, Table 1).

### 3.2. Relationship between HE4 Expression and Clinicopathological Parameters of Endometrial Cancer

HE4 expression in endometrial cancer was 76.32% at stage Ia, 91.30% at stage Ib, 71.43% at stage II, 96.43% at stage III, and 100.00% at stage IV, respectively, which showed an increasing tendency with increased clinical stage. Interestingly, the positive expression rate of HE4 at stage Ia was similar to that at stage II; the positive expression rate of HE4 at stage Ib was close to that at stage III or IV, indicating that the expression of HE4 was closely related to the invasion depth of the affected myometrium. Statistical analysis showed that there was a statistically significant difference in HE4 expression between stages III and Ia (*P*_Ia:III_ = 0.024), stages III and II (*P*_II:III_ = 0.035), stages IV and I (*P*_I:IV_ = 0.009), and stages III-IV and I-II (*P*_(I+II):(III+IV)_ = 0.007). The strongly positive expression rate of HE4 showed a similar trend. The strongly positive expression rate of HE4 in endometrial cancer was 31.57% at stage Ia, 56.52% at stage Ib, 28.57% at stage II, 71.42% at stage III, and 83.33% at stage IV, respectively, which showed an increasing tendency with increased clinical stage (*P*_Ia:III_ = 0.001, *P*_Ia:IV_ = 0.016, *P*_I:III_ = 0.008, *P*_I:IV_ = 0.047, *P*_II:III_ = 0.035, *P*_II:IV_ = 0.048, and *P*_(I+II):(III+IV)_ = 0.001).

HE4 expression in endometrial cancer was 72.72% in the highly differentiated group, 85.71% in the moderately differentiated group, and 92.31% in the poorly differentiated group, respectively. With a decrease in the degree of differentiation, the HE4 positive expression rate increased, and HE4 expression in the poorly differentiated group was significantly higher than that in the highly differentiated group (*P*_high:low_ = 0.024, *P*_high:middle_ = 0.348, and *P*_middle:low_ = 0.254). HE4 expression intensity was also closely related to the degree of differentiation. The strongly positive rate of HE4 expression was 34.62% in the highly differentiated group, 46.43% in the moderately differentiated group, and 57.69% in the poorly differentiated group, respectively, and showed no statistically significant differences between the groups (*P*_high:low_ = 0.186, *P*_high:middle_ = 0.696, and *P*_middle:low_ = 0.335).

HE4 positive expression rate in patients with lymph node metastasis was 89.66%, close to 84.93% in patients without lymph node metastasis (*P* = 0.532), whereas the strongly positive expression rate of HE4 in patients with lymph node metastasis (73.33%) was significantly higher than that in patients without lymph node metastasis (41.40%) (*P* = 0.002). HE4 positive expression rate was 82.14% in type I endometrial cancer and 87.84% in type II (*P* = 0.456); HE4 positive expression rates in endometrial adenocarcinoma, serous papillary carcinoma, clear cell carcinoma of the uterus, and other special pathological types were 91.84%, 71.43%, and 86.36%, respectively, and showed no significantly statistical difference between the groups (*P* > 0.05) (Figure 1, Table 2).

### 3.3. Prognosis Analysis

**Figure 2:**
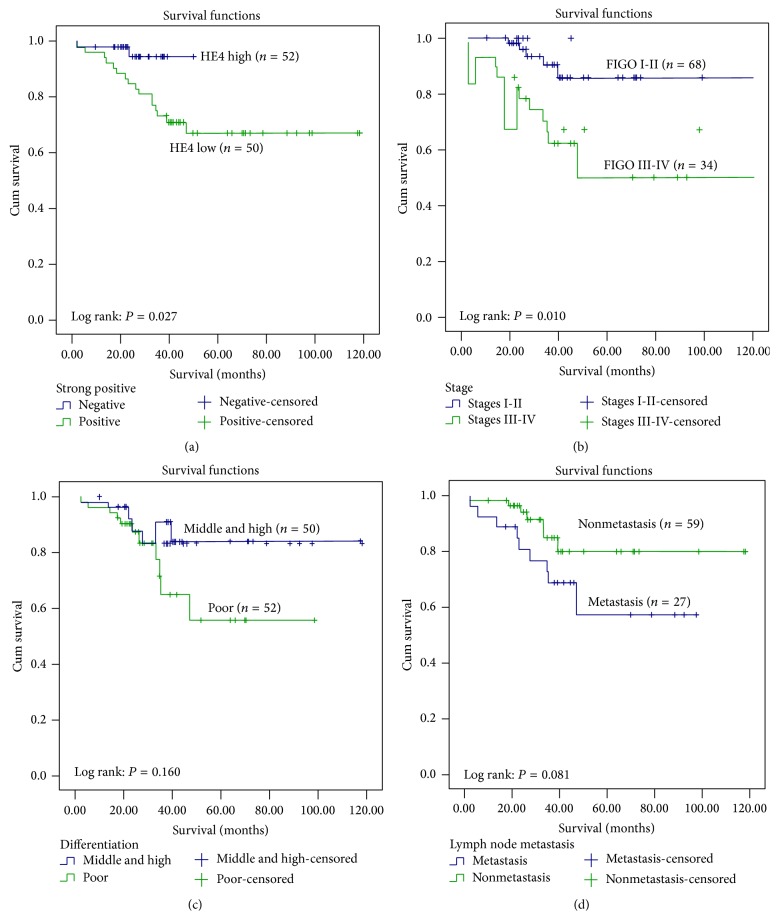
Comparison of survival rates. Curves of deaths stratified by (a) HE4 strong positive; (b) stage; (c) differentiation; and (d) lymphatic metastasis.

Up to May 2014, all patients were followed up for 9–116 months. Among 102 patients with endometrial cancer, 18 died due to tumor recurrence and metastasis. Kaplan-Meier survival analysis showed that endometrial cancer patients with strongly positive expression of HE4 had significantly higher mortality than those without strongly positive expression of HE4 (*P* = 0.027, Figure 2(a)); with an increase in endometrial cancer stage, mortality also showed a rising trend, and the mortality of endometrial cancer at FIGO stages III-IV was significantly higher than that in patients at FIGO stages I-II; the difference was statistically significant (*P* = 0.010, Figure 2(b)). Mortality in the poorly differentiated group and in patients with lymph node metastasis was also higher than that in the moderately or highly differentiated group and in patients without lymph node metastasis (*P* = 0.160, 0.081), but the difference was not statistically significant; therefore further follow-up is necessary (Figures 2(c) and 2(d)).

## 4. Discussion

In recent years, HE4 has been used as a tumor marker, and a number of serological tests have proved its early diagnostic value in epithelial ovarian tumors [3–5, 11]. The “2012 NCCN guidelines for the diagnosis and treatment of ovarian cancer” clearly indicate its clinical value as a tumor marker for epithelial ovarian cancer.

As the uterus and ovaries share the same embryonic origin, some pathological subtypes of ovarian cancer are endometrial adenocarcinomas, and both are malignant female reproductive system tumors. Thus, the determination of serum HE4 level in endometrial cancer patients has aroused wide attention. Moore et al. [12] determined multiple tumor markers in 156 healthy subjects and in 171 patients with endometrial cancer and found that HE4 expression at each stage of endometrial cancer was increased; the sensitivity of serum HE4 was higher than that of CA125. Angioli et al. [13] regarded serum HE4 concentration > 70 pmol/L as a quantitative indicator of endometrial cancer as this value showed the best sensitivity, specificity, and positive predictive value. Zanotti et al. [14] detected serum HE4 levels in 193 patients with endometrial carcinoma and in 125 healthy controls, and the results showed that a preoperative increase in HE4 is an independent prognostic factor for decreased overall survival, disease-free survival, and tumor progression-free survival in patients with endometrial cancer. In 2011, based on preliminary studies, Moore et al. [15] proposed the use of serum HE4 level to predict invasion depth of the myometrium to assess the necessity of preoperative lymph node dissection as a preoperative index. In 2011, Yang et al. [9] used an immunohistochemical method to detect the expression of HE4 in 31 cases of endometrial cancer, 19 cases of endometrial hyperplasia, and 20 cases of normal endometrial tissue, and the results showed that the positive expression rate of HE4 in the malignant group was significantly higher than that in the normal group and hyperplasia group and a statistically significant difference was observed between the groups. However, due to the small number of specimens and few subtypes in the cases (only four cases of special pathological types), the specimens did not explain the relationship between HE4 expression and lymph node metastasis. In addition, this study lacked a group with endometrial cancer FIGO IV stage and, in particular, lacked an atypical endometrial hyperplasia group, an important transitional pathological type.

In the present study, HE4 expression was detected on a larger scale: in 102 cases of endometrial cancer, 30 cases of atypical hyperplasia, and in 20 cases with normal endometrial tissue. The results showed that the positive expression rate of HE4 in endometrial cancer was significantly higher than that in atypical hyperplasia and normal endometrium (*P* < 0.05). The positive expression rate in atypical hyperplasia was significantly higher than that in the normal group, and the strongly positive expression rate in the severe hyperplasia group was significantly higher than that in the mild or moderate hyperplasia group. The HE4 expression level in normal, precancerous, and malignant tissues gradually increased as follows: normal tissues < precancerous lesions < malignant tissues, suggesting that HE4 may be involved in the development and progression of tumors. Li et al. [16] established endometrial cancer cell lines with HE4 overexpression and demonstrated that overexpression of HE4 enhanced the malignant behavior of cancer cells including proliferation, invasion, and colony formation. Real-time PCR showed that the mRNA and protein expression of HE4 in endometrial cancer tissue increased and immunofluorescence indicated that most of cells remained in the S phase of the cell cycle. These findings explain our results at the cellular and protein levels; however, the exact mechanism mediating the above-mentioned behavior requires further study.

Most endometrioid adenocarcinomas are estrogen-dependent (Type I), while other special histological types such as serous papillary carcinoma and clear cell carcinoma are nonestrogen-dependent (Type II). Our results show that HE4 is not correlated with pathological subtypes and estrogen-sensitivity; thus, unlike estrogen, to some extent, HE4 does not affect those at high risk of endometrial cancer, patients with obesity or diabetes and breast cancer patients on long-term administration of estrogen; that is [17], HE4 expression rate does not affect the pathological types of endometrial cancer and estrogen dependency. Therefore, we speculated that preoperative serum HE4 level cannot predict the histological subtypes of endometrial cancer, which is consistent with the findings of Bignotti et al. [18]. According to FIGO staging of endometrial cancer in 2009, cervical gland involvement is classified as stage I, rather than IIa. Stage II is defined as involvement of the cervical stroma, but without ectopic invasion. Cancerous tissues spread downwards to the stroma outside the cervical glands; however, because cervical tissue itself is much thinner than the myometrium, malignant tissue infiltration depth at stage II endometrial cancer may be less than that at stage I. Theoretically, the expression level of HE4 may be lower than that at stage I, which is in accordance with our study: with the exception of stage II, the positive expression and strongly positive expression rate of HE4 increased with increasing stage. The positive expression rate of HE4 in stage I endometrial cancer was higher than that at stage II, the strongly positive expression rate of HE4 at stages III and IV was significantly higher than that at stages I and II, the positive expression rate of HE4 at stages III and IV was higher than that at stage Ia, and the positive expression and strongly positive expression rate of HE4 in advanced endometrial cancer were significantly higher than that at earlier stages. It was noted that when stage FIGO Ia and Ib were compared, although no statistically significant difference was observed between the two, the HE4 expression rate and strongly positive expression rate differed greatly (91.30% versus 76.32%, 56.52% versus 31.57%, resp.), suggesting that the expression level of HE4 is not only related to the degree of ectopic metastasis but also associated with myometrial invasion depth. The larger the area and greater the depth of invasion, the more malignant cells are present and the higher the HE4 expression in corresponding tissues. Thus, as a secreted protein, more HE4 enters the blood, thereby increasing the peripheral blood concentration of HE4, which is consistent with findings in the literature [15, 16, 18]. However, a larger sample size in further studies is required to confirm these findings. As the degree of endometrial cancer differentiation decreased, the HE4 level increased, and the HE4 positive expression rate in the poorly differentiated group was significantly higher than that in the highly differentiated group, which demonstrated that HE4 expression in endometrial cancer is related to the degree of differentiation of the tumor. Prognostic analysis showed that mortality in patients with advanced endometrial cancer was significantly higher than that in patients at earlier stages and mortality in patients with high expression of HE4 was significantly higher than that in those with low expression, suggesting that HE4 may be involved in the recurrence, metastasis, and other adverse events of endometrial cancer. However, its mechanism needs to be clarified.

In recent studies, using overexpression and knockout of HE4 related genes, the malignant biological behavior such as cell adhesion, invasion, and proliferation was enhanced or inhibited in ovarian cancer cell lines. This was achieved through the EGFR-MAPK signal transduction pathway [19]. Following HE4 gene knockout, the phosphorylation levels of EGFR and Erk1/2 in ovarian cancer were affected; when HE4 was added to the cell culture, the phosphorylation levels of EGFR and Erk1/2 were restored. However, the mechanism involved is not yet clear. Lewis y antigen (a double-fucosylated oligosaccharide, located at the ends of many glycoproteins and glycolipids as a tumor-associated carbohydrate antigen) is part of the EGFR structure. Increased expression of Lewis y antigen activates EGFR and HER2/neu receptor tyrosine kinases, which further activate the PI3K/Akt and Raf/MEK/MAPK signal transduction pathways downstream of EGFR, resulting in accelerated transcription of HER2/neu genes in the nucleus and stimulation of DNA synthesis and ultimately promotes the cells to skip G1 phase into S phase, promoting cell proliferation and other kinds of malignant behavior [20]. Our previous studies confirmed the existence of the Lewis y structure on HE4, and ovarian cancer experiments showed that glycosylated HE4 had a stronger impact on cytobiology than nonglycosylated HE4 [to be published]. The modification of HE4 by Lewis y antigen enhanced tumor cell invasion, proliferation, adhesion, and other kinds of malignant behavior [10, 11, 16]. However, the mechanisms of HE4 mediating the occurrence and development of endometrial cancer require further study.

## 5. Conclusions

In summary, we conducted a large scale study and proved that the positive expression of HE4 in patients with endometrial cancer was significantly higher than that in patients with atypical hyperplasia and in those with normal endometrial tissue, which provides a preliminary theoretical reference for basic research on the role of HE4 in the development of endometrial cancer. However, whether HE4 mediates the biological behavior of endometrial cancer via the corresponding signal transduction pathways, as seen in ovarian cancer, thereby affecting the occurrence and development of endometrial cancer, requires further research.

## Figures and Tables

**Table 1:** HE4 expression in endometrial tissue.

Groups	Cases	−	+	++	+++	Positive cases	Positive rate (%)	Strong positive cases	Strong positive rate (%)

Endometrial cancer group	102	14	36	32	20	88	84.62^*^	52	55.98^*ϕ*^

Endometrial atypical hyperplasia group	30	10	14	6	0	20	66.67	6	20.00
Severe	10	2	4	4	0	8	80.00^**^	4	40.00^**^
Moderate	10	2	6	2	0	8	80.00^**^	2	20.00^*δ*^
Mild	10	6	4	0	0	4	40.00	0	0.00

Normal endometrium group	20	17	3	0	0	3	15.00	0	0.00
Secretory phase	10	8	2	0	0	2	20.00	0	0.00
Proliferative phase	10	9	1	0	0	1	10.00	0	0.00

Note: ^*^compared with the atypical hyperplasia group and normal group, *P* < 0.01; ^**^compared with the mild hyperplasia group, *P* < 0.05; compared with the normal group, *P* < 0.01; ^*ϕ*^compared with the atypical hyperplasia group, *P* < 0.05; compared with the normal group *P* < 0.01; ^*δ*^compared with the normal group, *P* < 0.05.

**Table 2:** HE4 expression in endometrial cancer with different clinical parameters.

Features		Cases	Positive cases	Positive rate (%)	Strong positive cases	Strong positive rate (%)	^*^ *P*	^**^ *P*

EC type	I	28	23	82.14	15	53.57	0.456	0.747
	II	74	65	87.84	37	50.00		

Pathological type	Endometrial adenocarcinoma	49	45	91.84	26	53.06		
	Uterine papillary serous carcinoma	21	15	71.43	8	38.10		
	Clear cell carcinoma	22	19	86.36	11	50.00		
	Mucous carcinoma	4	4	100.00	3	75.00	*P* > 0.05	*P* > 0.05
	Undifferentiated carcinoma	3	2	66.67	2	66.67		
	Squamous cell carcinoma	2	2	100.00	1	50.00		
	Small cell carcinoma	1	1	100.00	1	100.00		

FIGO stage	Ia Ib II III IV	38 23 7 28 6	29 21 5 27 6	76.32 91.30 71.43 96.43 100	12 13 2 20 5	31.57 56.52 28.57 71.42 83.33	*P*_Ia : III_ = 0.024 *P*_I : IV_ = 0.009 *P*_II : III_ = 0.035 *P*_(I+II) : (III+IV)_ = 0.007 Others *P* > 0.05	*P*_Ia : III_ = 0.001 *P*_Ia : IV_ = 0.016 *P*_I : III_ = 0.008 *P*_I : IV_ = 0.047 *P*_II : III_ = 0.035 *P*_II : IV_ = 0.048 *P*_(I+II) : (III+IV)_ = 0.001 Others *P* > 0.05

Differentiation level	High	22	16	72.72	9	34.62	*P*_high : low_ = 0.024 Others *P* > 0.05	*P* > 0.05
	Middle	28	24	85.71	13	46.43		
	Low	52	48	92.31	30	57.69		

Lymphatic metastasis	No	59	48	81.36	22	59.46	*P* = 0.532	*P* = 0.001
	Yes	27	26	96.30	21	77.78		
	No lymph node cleaning	16	14	87.50	9	56.25		

Note: ^*^comparison of HE4 positive rate in each group; ^**^comparison of strongly positive HE4 expression rate in each group.
