# Corrigendum to “Expression of HE4 in Endometrial Cancer and Its Clinical Significance”

**DOI:** 10.1155/2018/6795629

**Published:** 2018-09-12

**Authors:** Xiao Li, Yiping Gao, Mingzi Tan, Huiyu Zhuang, Jian Gao, Zhenhua Hu, Huimin Wang, Liancheng Zhu, Juanjuan Liu, Bei Lin

**Affiliations:** Department of Obstetrics and Gynecology, Shengjing Hospital of China Medical University, Shenyang, Liaoning 110004, China

In the article titled “Expression of HE4 in Endometrial Cancer and Its Clinical Significance” [1], there were errors in Figures 2(b) and 2(c), where some points were outside the survival curve due to a software error. The corrected version of Figure 2 is shown below. 

## Supplementary Materials

Supplementary Materials The raw data used in each version of SPSS.

## Figures and Tables

**Figure 2:**
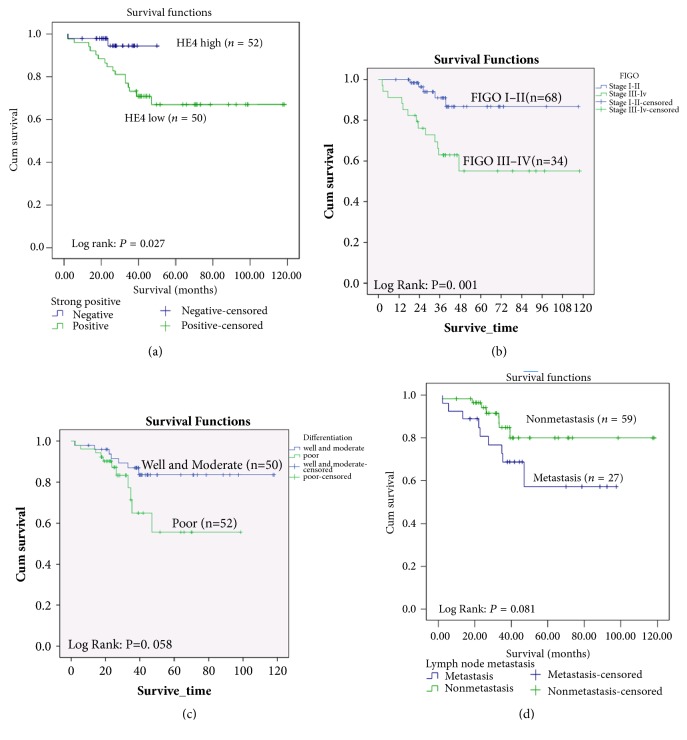
Comparison of survival rates. Curves of deaths stratified by (a) HE4 strong positive; (b) stage; (c) differentiation; and (d) lymphatic metastasis.
