# Supplementary figures and images for: Corrigendum to “Expression of HE4 in Endometrial Cancer and Its Clinical Significance”
Source: Biomed Res Int. 2018 Sep 12;2018:6795629. doi: 10.1155/2018/6795629 (PMC6157108; doi:10.1155/2018/6795629)

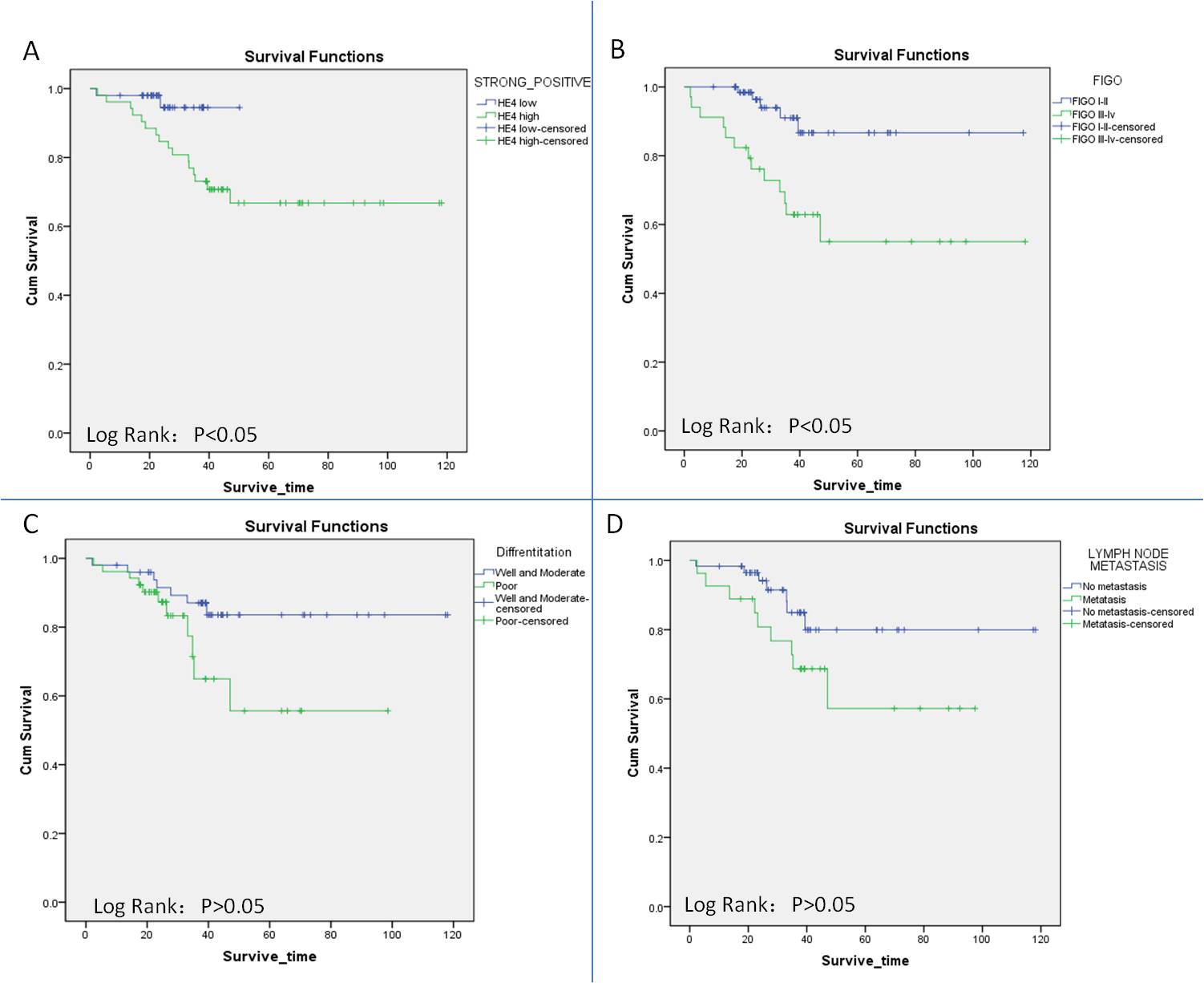

Supplement: Supplementary Materials — The raw data used in each version of SPSS. [file 6795629.f1.doc]
